# Efficient Pedestrian Detection at Nighttime Using a Thermal Camera

**DOI:** 10.3390/s17081850

**Published:** 2017-08-10

**Authors:** Jeonghyun Baek, Sungjun Hong, Jisu Kim, Euntai Kim

**Affiliations:** The School of Electrical and Electronic Engineering, Yonsei University, Seoul 120-749, Korea; jhyun25@yonsei.ac.kr (J.B.); imjune@yonsei.ac.kr (S.H.); jisukim2000@yonsei.ac.kr (J.K.)

**Keywords:** pedestrian detection, far-infrared sensor, thermal-position-intensity-histogram of oriented gradient

## Abstract

Most of the commercial nighttime pedestrian detection (PD) methods reported previously utilized the histogram of oriented gradient (HOG) or the local binary pattern (LBP) as the feature and the support vector machine (SVM) as the classifier using thermal camera images. In this paper, we propose a new feature called the thermal-position-intensity-histogram of oriented gradient (TPIHOG or TπHOG) and developed a new combination of the TπHOG and the additive kernel SVM (AKSVM) for efficient nighttime pedestrian detection. The proposed TπHOG includes detailed information on gradient location; therefore, it has more distinctive power than the HOG. The AKSVM performs better than the linear SVM in terms of detection performance, while it is much faster than other kernel SVMs. The combined TπHOG-AKSVM showed effective nighttime PD performance with fast computational time. The proposed method was experimentally tested with the KAIST pedestrian dataset and showed better performance compared with other conventional methods.

## 1. Introduction

For the commercialization of the advanced driver assistance system (ADAS), the most important factors are reliability and robustness, and pedestrian detection (PD) is certainly one of the ADAS functions that require high reliability and robustness. For a robust and reliable PD, reasonable performance even in the nighttime is important because more than half of pedestrian-related accidents occur in the nighttime, even though the volume of traffic is much less than in the daytime [1,2].

For effective nighttime PD, most studies used a thermal camera sensor because it visualizes objects using the infrared (IR) heat signature and it does not depend on lighting conditions. Among several types of thermal cameras, the far infrared (FIR) sensor is commonly used for PD in the nighttime because thermal radiation from pedestrian peaks in the FIR spectrum [3]. Compared with visible images, FIR images are robust against illumination variation but are significantly affected by weather because FIR sensors capture temperature changes in the output images. For example, pedestrians appear brighter than the background in cold days while they appear darker in hot days [1]. Furthermore, FIR images only contain a single channel of intensity information; thus, information on these images is not as detailed as that of visible images.

Similar to PD using visible images, PD using FIR images also consists of two steps: feature extraction and classification. In the feature extraction step, the features developed for daytime PD can also be used for nighttime PD. For example, local binary patter (LBP) [4] and its variations, such as the HOG-LBP [1,5], center-symmetric LBP (CSLBP) [6], and oriented CSLBP (OCSLBP) [7] were also proposed as daytime PD features. However, the LBP-based features have only orientation information of pixel intensity; therefore, they are sensitive to lighting conditions. On the other hand, there are some methods that use the shape of pedestrians as features. Dai et al. [8] utilized the joint shape and appearance cue to find the exact locations of pedestrians. Wang et al. [9] extracted the features using a shape describer and Zhao et al. [3] proposed the shape distribution histogram (SDH). These shape-based features simply used only pixel intensity information and employed background subtraction methods for fixed camera images. Therefore, shape-based features are not suitable for vehicle environment where complex background is not fixed.

As a robust feature for pedestrian detection, the histograms of oriented gradient (HOG) [10] is one of the most popular PD features and its variations have been proposed [11]. Co-occurrence HOG (CoHOG) is one of the extensions of the HOG and it utilizes pair of orientations for computing histogram feature [12]. N. Andavarapu et al. [13] proposed weighted CoHOG (W-CoHOG) that considers gradient magnitude factor to extracting CoHOG. Spatio-temporal HOG (SPHOG), which contains motion information, is proposed for image sequences with fixed camera [14]. Scattered difference of directional gradient (SDDG) that extracts local gradient information along the certain direction is also proposed for IR images [15]. Kim et al. proposed position-intensity HOG (PIHOG or πHOG) that includes not only HOG but also the detail position and intensity information for vehicle detection [16]. Theses HOG based features utilize only the gradient information based on color images or do not consider the thermal intensity information which is important cue for pedestrian detection in nighttime.

To address these problems of conventional features, we propose a thermal position intensity HOG (TPIHOG or TπHOG). The TπHOG is the extended version of πHOG and it is applied for pedestrian detection in nighttime. Unlike the πHOG, the proposed TπHOG has thermal intensity information and can be computed more simply than πHOG.

With respect to the classification, the linear support vector machine (linear SVM) is widely used as classifier in many studies, such as in [17,18,19] because it is fast and has reasonably good performance. The kernel SVM has better classification performance than the linear SVM but requires a longer computation time owing to kernel expansion [20,21,22]. However, the additive kernel SVM (AKSVM) has better performance than the linear SVM and also has a classification speed comparable with the linear SVM [20,23,24]. Recently, deep learning has also been applied to object detection system.

Kim et al. utilize convolutional neural network (CNN) for nighttime PD using visible images [25]. Liu et al. and Wagner et al applied fusion architectures to CNN which fuse the visible channel feature and thermal channel features for multispectral PD [26,27]. Cai et al. generates the candidates using saliency map and used deep belief network (DBN) as a classifier for vehicle detection in nighttime [28]. John et al. used Fuzzy C-means clustering for generating candidates and CNN for verification for PD in thermal images [29]. However, the deep learning based method requires dataset with large clean annotations and training procedure, which takes too much time to converge [30]. In addition, GPU is necessary for the training of deep learning, but it is not suitable for implement autonomous system because the system needs to be embedded system [31].

In this study, we propose a combination of the TπHOG and the AKSVM (TπHOG-AKSVM) to achieve improved performance for nighttime PD in terms of PD performance compared with conventional methods.

The remainder of this paper is organized as follows. In Section 2, some background about the πHOG is presented. Details of the proposed TπHOG-AKSVM are presented in Section 3. Section 4 presents experimental results and discussions, and conclusions are drawn in Section 5.

## 2. Preliminary Fundamentals

### Position Intensity Histogram of Oriented Gradient (πHOG)

The HOG is defined as the histogram of the magnitude sum for gradient orientations in a cell and it is widely used as an effective feature for PD or vehicle detection (VD). The HOG feature, however, has the limitation that information on gradient position in the cell is lost and the pixel intensity information is not used. Recently, the πHOG has been proposed to address this problem and shows better detection performance than HOG for vehicle detection [16]. The πHOG contains not only the HOG but also additional information about gradient Position and pixel Intensity. The πHOG consists of three parts: the Position (**P**) part, the Intensity (**I**) part, and the conventional HOG. The **P** part of the πHOG is extracted by computing the average position of each orientation bin. That is, if θ(x,y,c) denotes the orientation of the gradient at position (x,y) of the c-th cell and the orientation bin of the gradient B(x,y,c) is defined by

(1) $$B(x,y,c)=\left\lceil \frac{T\theta (x,y,c)}{2\pi }\right\rceil ,\;\;\;\left(0\leq \theta \left(x,y,c\right)<2\pi \right)$$

where T is the number of bins and B(x,y,c)∈{1, ⋯ , T}, then the averages of x and y positions of the d-th bin (d∈{1, ⋯ , T}) in the c-th cell are defined by

(2) $$\begin{array}{l} M_{x,d}^{c}=\frac{\sum_{x=1}^{c_{s}}\sum_{y=1}^{c_{s}}xI\left[B\left(x,y,c\right)=d\right]}{\sum_{x=1}^{c_{s}}\sum_{y=1}^{c_{s}}I\left[B\left(x,y,c\right)=d\right]}\\ M_{y,d}^{c}=\frac{\sum_{x=1}^{c_{s}}\sum_{y=1}^{c_{s}}yI\left[B\left(x,y,c\right)=d\right]}{\sum_{x=1}^{c_{s}}\sum_{y=1}^{c_{s}}I\left[B\left(x,y,c\right)=d\right]} \end{array}$$

where $c_{s}$ denotes the cell size and I(⋅) is the binary function which returns 1 if the input argument is true and 0 if the argument is false. Then the **P** part of the c-th cell in πHOG is $P_{c}=[M_{x}^{c},\;M_{y}^{c}]$, where $M_{x}^{c}=\left[M_{x,1}^{c},\;\cdots \;,\;M_{x,T}^{c}\right]$ and $M_{y}^{c}=\left[M_{y,1}^{c},\;\cdots \;,\;M_{y,T}^{c}\right]$.

The **I** part of the πHOG can be defined in terms of the pixel intensity of vehicle images. There are a variety of shapes and sizes of vehicles, but all types of vehicles have low intensity values in some common areas, such as tires and bottom of the vehicles. Using this knowledge, the intensity invariant region (IIR) was proposed in [16]. The IIR is defined as the region of pixels in which the corresponding standard deviation is relatively low. Then, the **I** part is defined as the summation of standard normal deviation values in the IIR. A detailed procedure for extracting the **I** part of the πHOG is explained as follows. When a set of positive vehicle images $V^{+}=\left\{s_{1},\;s_{2},\;\cdots ,\;s_{N_{v}}\right\}$ is given, where $N_{v}$ is the number of the training images in $V^{+}$; $s={\left[s_{1},\;\;s_{2},\;\cdots \;,\;\;s_{N}\right]}^{T}\in {ℜ}^{N}$ is the vehicle image and all the images in $V^{+}$ are resized to images of same size and aligned; $s_{j}$ is the intensity value of jth pixel value of s and N is the image size, the mean and standard deviation of the vehicle images are defined as

(3) $$M=\frac{1}{N_{v}}\sum_{i=1}^{N_{v}}s_{i}={\left[m_{1},\;\;m_{2},\;\cdots \;,\;\;m_{N}\right]}^{T}$$

(4) $$\sigma =\sqrt{\frac{1}{N_{v}}\sum_{i=1}^{N_{v}}\left(s_{i}-M\right)\circ \left(s_{i}-M\right)}={\left[\sigma_{1},\;\;\sigma_{2},\;\cdots \;,\;\;\sigma_{N}\right]}^{T}$$

where ∘ denotes component-wise multiplication. In Equation (4), low σ means that the corresponding region has similar intensity values over all types of vehicles including sedans, trucks or sport utility vehicles (SUVs). Therefore, the region with low standard deviation σ can be used as a distinctive cue for classifying vehicles. This region was defined as IIR and a new feature was extracted from the IIR [19]. To determine the IIR, the values of σ are divided into M intervals and the binary mask $U_{k}$ (k=1,2,...,M) is constructed as

(5) $$U_{k}=\left\{j|\xi_{k}\leq \sigma_{j}\leq \xi_{k+1},\;\;j=1,2,\cdots ,N\right\}$$

where $\xi_{k}$ is

(6) $$\xi_{k}=\left(k-1\right)\times \left\lceil \frac{N}{M}\right\rceil \mathrm{th}\;\mathrm{smallest}\;\mathrm{value}\;\mathrm{of}.$$

Finally, the I part of the πHOG is the feature for the IIR region masked by $U_{k}$ of standard normal deviate image z. That is, if the test image $s\in {ℜ}^{N}$ is given, then z is computed by

(7) $$z=\left(\frac{1}{\sigma }\right)\circ \left(s-M\right)\in {ℜ}^{N}$$

and the **I** part is defined by

(8) $$I=[h_{1},...,h_{k}]$$

where

(9) $$h_{k}=\frac{1}{\left|U_{k}\right|}\sum_{j\in U_{k}}z_{j}$$

**Figure 1:**
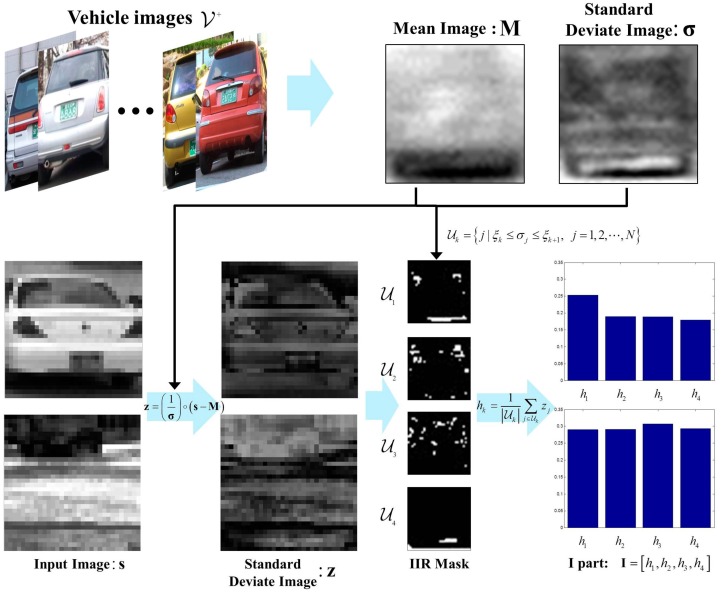
Examples of computing **I** part using 4 IIR masks [16].

Figure 1 is an example of computing **I** part using 4 IIR masks from testing images.

Finally, the πHOG is defined as a concatenation of the three parts, the HOG, the **P** part and the **I** part.

## 3. Proposed Method

**Figure 2:**
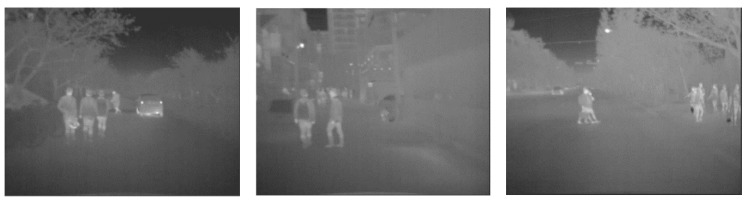
Examples of thermal images in KAIST dataset.

Figure 2 shows some examples of pedestrians in thermal images. As shown in the figure, pedestrian detection using a thermal sensor is quite different from PD using a visible sensor owing to the characteristics of the thermal images.

In thermal images, pedestrians appear brighter than the background and they do not include any color information, only silhouettes. In addition, their intensities vary according to changes in the weather because the thermal sensors visualize temperature radiation from the objects in the images. Therefore, it is important to extract features that can reliably capture pedestrian silhouette under various weather conditions in thermal images.

In previous works [1,5], the HOG was popularly used for nighttime pedestrian detection because it captures the appearance of pedestrians by stacking gradient information. However, in this paper, a new feature called the TπHOG is proposed to improve nighttime PD performance of the HOG. The proposed TπHOG is based on the πHOG [16] and it is developed so that the TπHOG has more distinctiveness than the HOG when thermal sensors are used. The TπHOG includes not only thermal gradient information but also its locations and thermal intensities. The TπHOG is not a simple application of the πHOG to thermal images, but it is redesigned to handle PD problems in thermal images.

In addition, instead of the linear SVM, the additive kernel support vector machine (AKSVM) is used as a classifier to enhance the detection performance, as well as the detection time.

### 3.1. Thermal Position Intensity Histogram of Oriented Gradient (TπHOG)

The πHOG takes longer time and thus, computationally more expensive than the HOG since the πHOG requires additional pixel-wise computation to compute the mean of pixel locations. However, the pixel-wise computation in the πHOG is not suitable for commercial PD because it requires real time operation. Thus, in the proposed TπHOG, the cell-wise approach, and not the pixel-wise approach, was adopted to reduce the computational time. Since the cell values have already been computed in extracting the HOG, less computation is required to compute the TπHOG compared with the conventional HOG.

Furthermore, unlike the **I** part based on the IIR in [16], the **I** part in this paper was increased such that it has the same size as the orientation channel of the HOG. This is because the **I** part in the original work [16] used only 4 values from the 4 IIR masks as features and it had relatively small effects on the PD performance compared with the **P** or the HOG parts.

The TπHOG consists of four parts: the **T** part, the Position (**P**) part, the Intensity (**I**) part, and the conventional HOG. In the conventional HOG, we used the HOG of [32] which has 27 gradient channels (18 signed orientations, 9 unsigned orientations) and 4 gradient energy channels using different normalization methods. A detailed description of these four parts is presented in following subsections.

#### 3.1.1. **T** Channel Part

**Figure 3:**
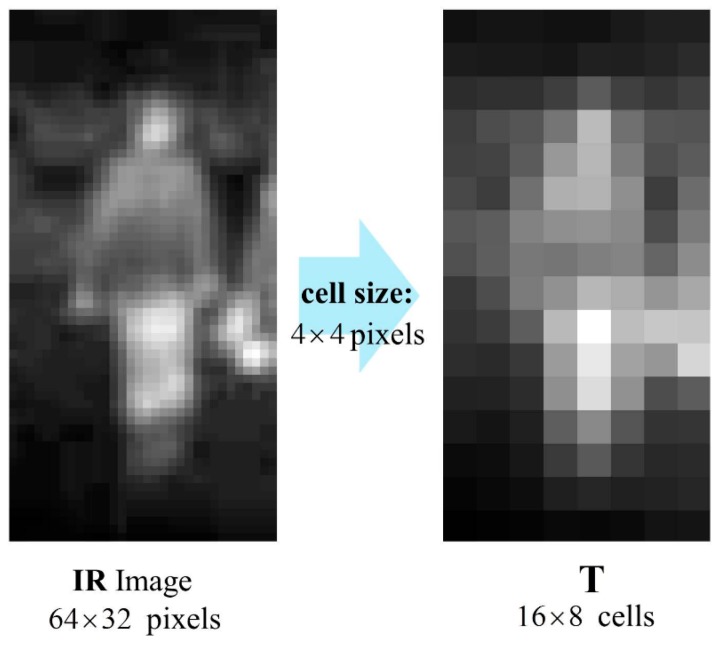
Example of IR image and its **T** channel.

For the first part of the TπHOG, we used the **T** channel proposed in [33]. The **T** channel is defined as an aggregated version of a thermal image. For example, given 64 × 32 IR images and 4 × 4 cell size, the **T** channel has 16 × 8 cells and the value in each cell is the sum of pixel intensities within the cell. Figure 3 shows an example of an IR image and its **T** channel.

Unlike the visible image, the pedestrians have higher pixel intensity values than backgrounds in IR images. Thus, **T** channel that consists of aggregations of IR intensities can play an important role in classifying pedestrians from other objects.

#### 3.1.2. Position Part

**Figure 4:**
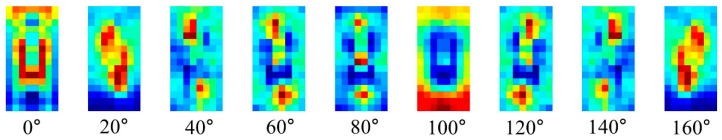
Example of HOG channels with 9 orientations.

In the **P** part of the TπHOG, cell locations of the gradients and not pixel locations, are used unlike in the πHOG. In the feature implementation, the HOG consists of multiple orientation channels and each channel contains the bin value for the corresponding orientation of a cell histogram. In Figure 4, shown are the examples of HOG that has 16 × 8 cells with 9 gradient orientations.

**Figure 5:**
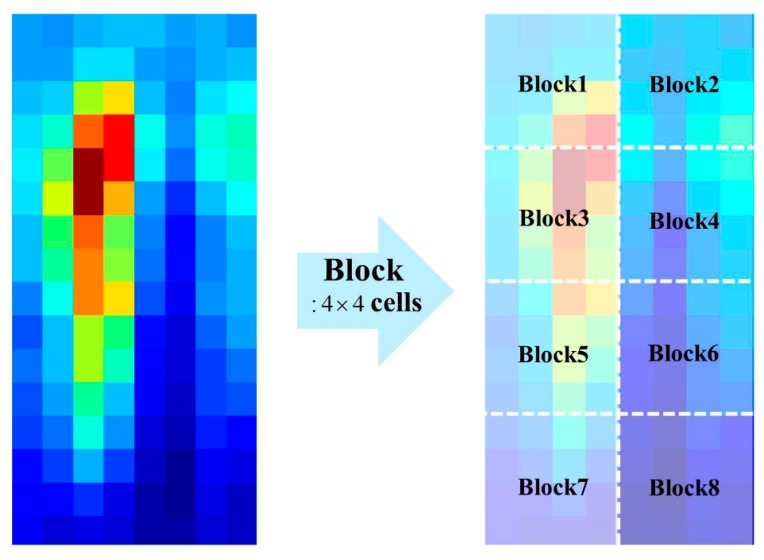
Example of dividing HOG into 8 blocks.

In Figure 4, the values in each channel denote the bin values of cell histogram for the corresponding orientation. For example, the first channel of the HOG contains the first bin value of the cell histogram. In computing the **P** part of the TπHOG, we divide the HOG cells into several blocks as shown in Figure 5.

**Figure 6:**
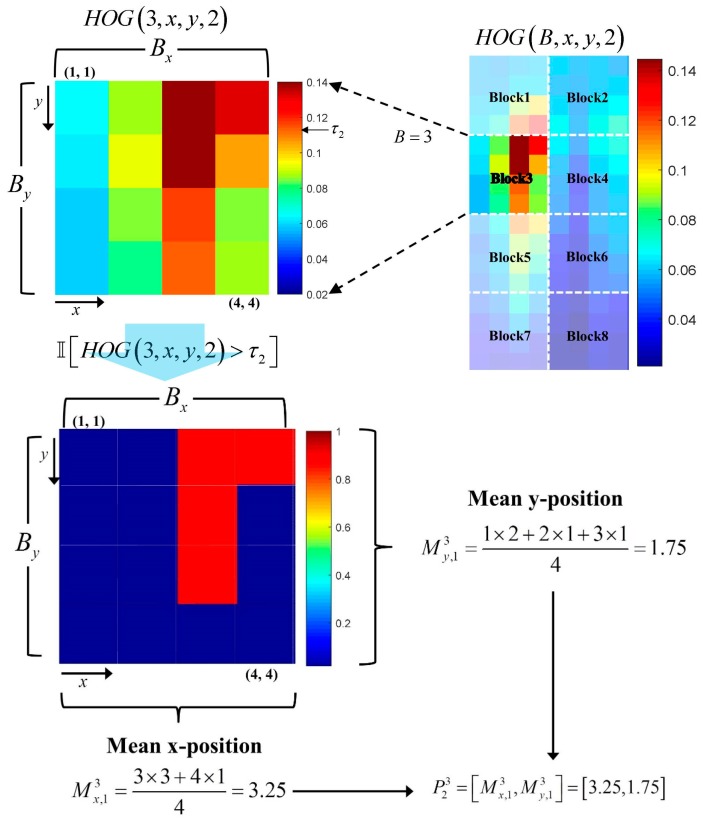
Example of **P** part extraction.

The **P** part is defined as the (x,y) location in which each orientation component exists in a block. Assuming that HOG(B,x,y,d) is the value of a cell located at (x,y) of the Bth block in the dth orientation channel, then the **P** part is defined by

(10) $$P_{d}^{B}=\left[M_{x,d}^{B},M_{y,d}^{B}\right]$$

where

(11) $$\begin{array}{l} M_{x,d}^{B}=\frac{\sum_{x=1}^{B_{s}}\sum_{y=1}^{B_{s}}xI\left[HOG\left(B,x,y,d\right)>\tau_{d}\right]}{\sum_{x=1}^{B_{s}}\sum_{y=1}^{B_{s}}I\left[HOG\left(B,x,y,d\right)>\tau_{d}\right]}\\ M_{y,d}^{B}=\frac{\sum_{x=1}^{B_{s}}\sum_{y=1}^{B_{s}}yI\left[HOG\left(B,x,y,d\right)>\tau_{d}\right]}{\sum_{x=1}^{B_{s}}\sum_{y=1}^{B_{s}}I\left[HOG\left(B,x,y,d\right)>\tau_{d}\right]} \end{array}$$

where $\tau_{d}$ is the threshold for each orientation. Figure 6 shows an example of the computation of the two values for $P_{2}^{3}$, the 3rd block in the second orientation channel, when the block size is 4×4 cells with 9 orientations.

In Figure 6, only the cells with values of that exceed the threshold $\tau_{2}$ are used to compute the **P** part $P_{2}^{3}$ of the TπHOG. Similarly, the **P** part contains location information of each orientation channel and is computed by

(12) $$P=\left[P_{1},\;\cdots \;,P_{9}\right]$$

where $P_{d}=\left[P_{d}^{1},\;\cdots \;,\;P_{d}^{8}\right]$.

**Figure 7:**
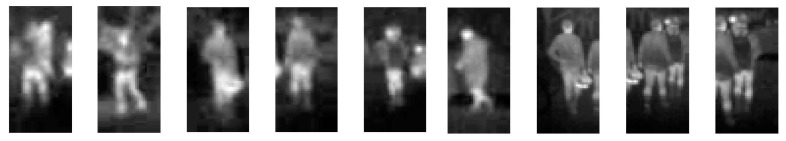
Examples of pedestrian IR images in KAIST pedestrian dataset [33].

**Figure 8:**
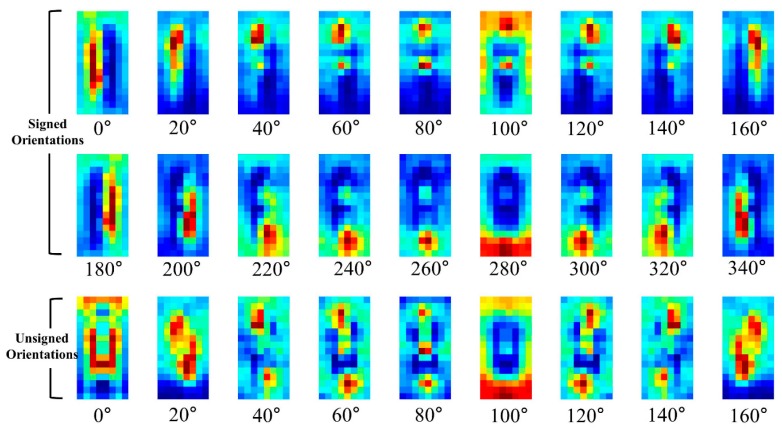
Average gradient channels of HOG for pedestrians.

**Figure 9:**
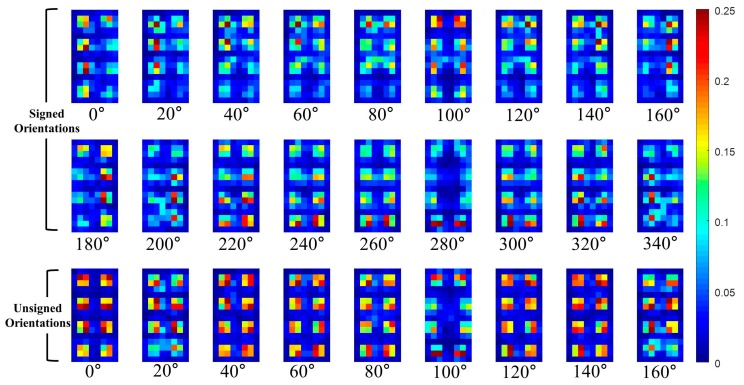
Average of **P** parts of TπHOG for pedestrians.

For the sake of better understanding of **P** part, the HOG and **P** part are visualized in Figure 7, Figure 8 and Figure 9. Shown in Figure 7 are the examples of pedestrians IR images and they are adopted from KAIST pedestrian dataset [33].

The average HOG channels for training pedestrian data are visualized in Figure 8. The average channels are computing using 64 × 32 cropped IR images of 2244 pedestrians. In the figure, the first two rows indicate the HOGs for signed 18 orientations while the third row indicates the HOGs for unsigned 9 orientations.

In Figure 8, the closer the cell is to the red color, the higher the corresponding HOG. As shown in the figure, pedestrians have specific cell parts that have relatively high values for each orientation channel. The conventional HOG uses only these values as the feature but the TπHOG also uses the cell locations of the orientations as well for nighttime PD.

**Figure 10:**
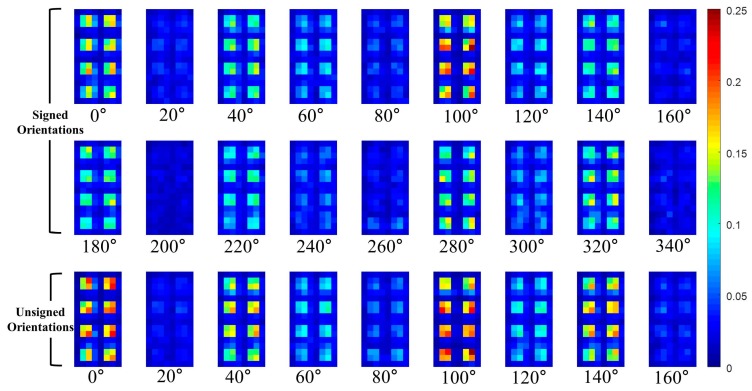
Average of **P** parts of TπHOG for non-pedestrians.

In this paper, the **P** part of the TπHOG is extracted from non-overlapped blocks that comprising of 4 × 4 cells. For example, assuming the HOG has 31 channels (18 signed orientations, 9 unsigned orientations, 4 different normalizations), the size of the HOG is 16 × 8 × 31 cells and it has 4 × 2 × 31=248 blocks. The **P** part $P_{o}^{B}=\left[M_{x,o}^{B},M_{y,o}^{B}\right]$ of the TπHOG is extracted from each block and the **P** part has additional 496 values. Figure 9 and Figure 10, show the visualization of the average $P=\left[P_{1},\;\cdots \;,P_{9}\right]$ of the TπHOG for pedestrians and non-pedestrians, respectively. In Figure 9 and Figure 10, the average **P** parts are computed for 2244 pedestrian images and 5000 non-pedestrian images, respectively. All the images are 64 × 32 cropped IR images.

As shown in Figure 9 and Figure 10, the average **P** parts of the pedestrians are focused on a couple of cell locations for each block. In particular, the average **P** parts of the pedestrians are mostly larger than those of the non-pedestrians except for unsigned orientation of $0^{\circ }$ and $100^{\circ }$. Instead, the average **P** parts of the non-pedestrians usually have lower values than those of pedestrians. Further, in the orientations of $20^{\circ }$ and $160^{\circ }$, the **P** parts of non-pedestrians have the values close to 0 and they are not included in computation of **P** parts. This difference between the two classes provides the TπHOG with discriminatory power for robust PD compared with the HOG.

#### 3.1.3. Intensity Part

The conventional **I** part of the πHOG is defined as a partial pixel-wise sum of the standard deviate image [16] within the IIR. Thus, the evaluation of the **I** part requires pixel-wise computation; however, it is obviously computationally expensive for real-time application. Furthermore, the conventional **I** part in [16] is 4 dimensions long and too short compared with 3968 (16  ×  8  ×  31) dimensions of the HOG, thereby producing minimal effect on the PD performance. In this paper, a modified version of the **I** part is developed for the PD in thermal images. Rather than using IIR masks, the new **I** part is directly computed from a normal standard deviate image of the set of **T** channels which are computed from training pedestrian data (64  ×  32 cropped IR images of pedestrians). Therefore, the feature length of the **I** part is the same as that of the **T** channel. That is, given the **T** channel set of pedestrian images $T^{+}=\left\{T_{1},\;T_{2},\;\cdots ,\;T_{N_{p}}\right\}$ where the superscript ‘+’ means the positive pedestrian samples, $N_{p}$ is the number of the T channels in $T^{+}$ and $T\in {ℜ}^{16\times 8}$. $M_{T}$ and $\sigma_{T}$ are the mean and standard deviation of $T^{+}$, respectively, and the **I** part for a testing **T** channel $s_{T}$ is computed by

(13) $$I_{T}=\left|\left(\frac{1}{\sigma_{T}}\right)\circ \left(s_{T}-M_{T}\right)\right|$$

**Figure 11:**
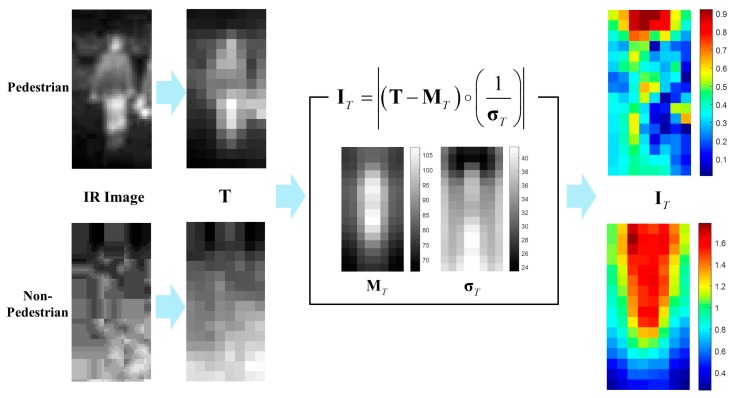
Example of computing **I** part from testing images.

How to compute the **I** part from both pedestrian and non-pedestrian testing images is summarized in Figure 11.

Shown in Figure 11 is the average of **I** parts for pedestrians and non-pedestrians. The images are 64 × 32 cropped IR images of testing dataset of KAIST pedestrians Dataset.

**Figure 12:**
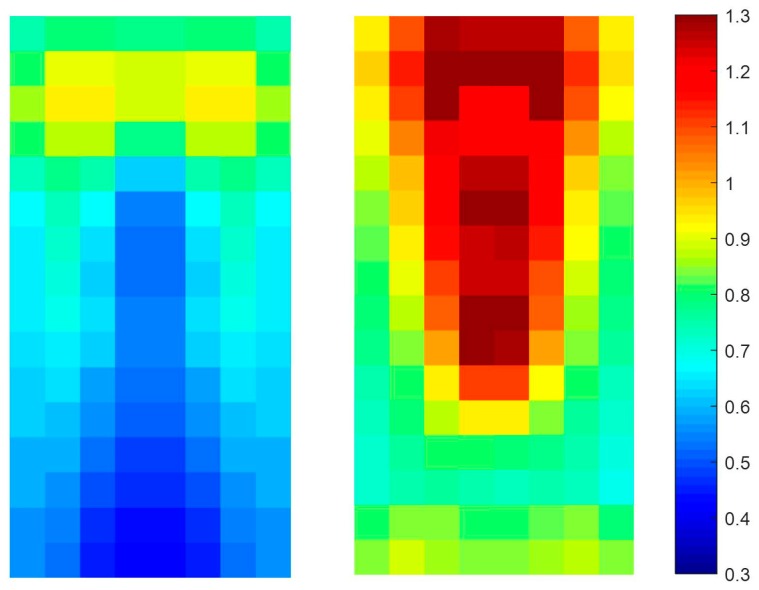
Average **I** part for testing images(**Left**: pedestrian, **Right**: non-pedestrian).

As shown in Figure 12, most of the average **I** parts for pedestrian images are less than 1. In particular, the parts corresponding to the lower body are less than 0.7. On the other hand, the average **I** parts for non-pedestrian generally are larger than 0.7 and the parts corresponding to the upper body have the values larger than 1. This difference provides the **I** parts with strong discriminatory power between pedestrians and non-pedestrians. Further, the extraction of the **I** part is a cell-wise computation and does not require the additional computation for developing the histogram; therefore, the proposed **I** part is computationally more efficient than that of the conventional πHOG [16].

### 3.2. Additive Kernel SVM (AKSVM)

The SVM is one of the popular binary classifiers used in object detection in computer vision. Given the training set $S={\left\{\left(x^{\left(i\right)},y^{\left(i\right)}\right)\right\}}_{i=1}^{L}$ with L samples, the SVM is trained to classify input data $x^{\left(i\right)}$ as positive ($y^{\left(i\right)}=1$) class or negative ($y^{\left(i\right)}=-1$) class where $x^{\left(i\right)}={\left[x_{1}^{\left(i\right)},x_{2}^{\left(i\right)},...,x_{N}^{\left(i\right)}\right]}^{T}\in {ℜ}^{N}$ and $y^{\left(i\right)}\in \left\{-1,+1\right\}$. If input data $x^{\left(i\right)}$ is mapped to a higher dimensional feature space as ϕ(⋅) then the decision function of the SVM is defined by

(14) $$f\left(x\right)=w^{T}\phi \left(x\right)+b$$

where $\phi \left(x\right)\in {ℜ}^{D}$, D≫N, $w\in {ℜ}^{D}$ is the weight and b∈ℜ is the bias. The SVM is trained by finding optimal solutions of w and b which maximizes the margin between the two classes. It can also be trained in dual space using the kernel trick with $\kappa \left(x^{\left(i\right)},x^{\left(j\right)}\right)=\phi \left(x^{\left(i\right)}\right)\cdot \phi \left(x^{\left(j\right)}\right)\in ℜ$. After training the SVM in dual space, the decision function (11) can be evaluated by

(15) $$\begin{array}{ll} f\left(x\right) & =\sum_{i=1}^{L}\alpha^{\left(i\right)}y^{\left(i\right)}\kappa \left(x^{\left(i\right)},x\right)+b\\ & =\sum_{i\in SV}\alpha^{\left(i\right)}y^{\left(i\right)}\kappa \left(x^{\left(i\right)},x\right)+b \end{array}$$

where $SV=\{i|\alpha^{\left(i\right)}>0\}$ denotes a set of support vectors. If the κ(⋅) of Equation (15) is nonlinear, the kernel SVM performs better than the linear SVM in classification. However, it requires the high computation and memory resource owing to the kernel computation with its support vectors for every testing.

However, the additive kernels (AK) enable fast computation of the decision function, while maintaining the robust performance of the kernel SVM [23,24]. The AK is defined as the kernel that can be decomposed into a summation of dimension-wise components and it is represented by $\kappa \left(x,z\right)=\sum_{n=1}^{N}\kappa_{n}\left(x_{n},z_{n}\right)$ where $x=\left\{x_{1},x_{2},...,x_{N}\right\}\in {ℜ}^{N}$ and $z=\left\{z_{1},z_{2},...,z_{N}\right\}\in {ℜ}^{N}$. Various AKs have been reported and they include the linear kernel $\kappa_{LIN}$, intersection kernel $\kappa_{IK}$, the generalized intersection kernel $\kappa_{GIK}$ and the $\chi^{2}$ kernel $\kappa_{\chi^{2}}$ defined as

(16) $$\kappa_{IK}\left(x,z\right)=\sum_{n=1}^{N}\min\left(x_{n},z_{n}\right)$$

(17) $$\kappa_{GIK}\left(x,z\right)=\sum_{n=1}^{N}\min\left({\left|x_{n}\right|}^{2},{\left|z_{n}\right|}^{2}\right)$$

(18) $$\kappa_{\chi^{2}}\left(x,z\right)=\sum_{n=1}^{N}\frac{2x_{n}z_{n}}{x_{n}+z_{n}}$$

**Figure 13:**
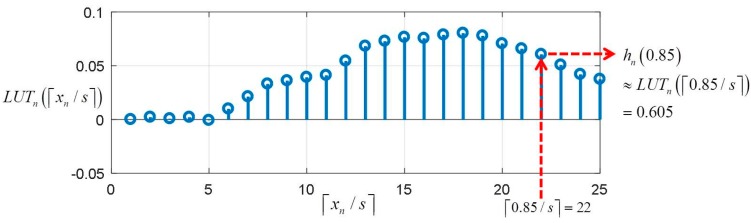
Example of retrieving value of $h_{n}\left(x_{n}\right)$ from $LUT_{n}$ for $x_{n}=0.85$.

The decision function of the SVM in Equation (15) with the AK can be represented by

(19) $$\begin{array}{ll} f_{AK}\left(x\right) & =\sum_{i=1}^{L}\alpha^{\left(i\right)}y^{\left(i\right)}\kappa \left(x^{\left(i\right)},x\right)+b\\ & =\sum_{i=1}^{L}\alpha^{\left(i\right)}y^{\left(i\right)}\sum_{n=1}^{N}\kappa_{n}\left(x_{n}^{\left(i\right)},x_{n}\right)+b\\ & =\sum_{n=1}^{N}\left\{\sum_{i=1}^{L}\alpha^{\left(i\right)}y^{\left(i\right)}\kappa_{n}\left(x_{n}^{\left(i\right)},x_{n}\right)\right\}+b\\ & =\sum_{n=1}^{N}h_{n}\left(x_{n}\right)+b \end{array}$$

where

(20) $$\begin{array}{ll} h_{n}\left(x_{n}\right) & =\sum_{i=1}^{L}\alpha^{\left(i\right)}y^{\left(i\right)}\kappa_{n}\left(x_{n}^{\left(i\right)},x_{n}\right)\\ & =\sum_{i\in SV}\alpha^{\left(i\right)}y^{\left(i\right)}\kappa_{n}\left(x_{n}^{\left(i\right)},x_{n}\right) \end{array}$$

and $h_{n}\left(x_{n}\right)$ is a one-dimensional function of $x_{n}\in ℜ$. In Equation (20), the $\alpha^{\left(i\right)}$, $y^{\left(i\right)}$ are given; therefore the output of $h_{n}\left(\cdot \right)$ can be pre-computed for all possible input data $x_{n}\in ℜ$ and computed output values are stored in look-up-table (LUT) for each $h_{n}\left(\cdot \right)$. Assuming the $LUT_{n}\in {ℜ}^{N_{L}}$ is the LUT that consists of sampled $N_{L}$ values from $h_{n}\left(\cdot \right)$, $h_{n}\left(x_{n}\right)$ of Equation (20) can be simply approximated as

(21) $$h_{n}\left(x_{n}\right)\approx LUT_{n}\left(\left\lceil x_{n}/s\right\rceil \right)$$

where $s=1/N_{L}$ is the sampling interval on $x_{n}$. Figure 13 shows an example of of retrieving value of $h_{n}\left(x_{n}\right)$ from $LUT_{n}$. In Figure 13, the size of $LUT_{n}$ is $N_{L}=25$ and the values of $LUT_{n}$ are sampled from $h_{n}\left(\cdot \right)$ with sample interval s=0.04.

Therefore, using the LUTs of $h_{n}\left(\cdot \right)$, testing of Equation (20) can be simplify carried out as the summation of values taken from the $LUT_{n}$s without kernel computation as

(22) $$f_{AK}\left(x\right)\approx \sum_{n=1}^{N}LUT_{n}\left(\left\lceil x_{n}/s\right\rceil \right)+b$$

### 3.3. TπHOG-AKSVM for Nighttime PD

**Figure 14:**
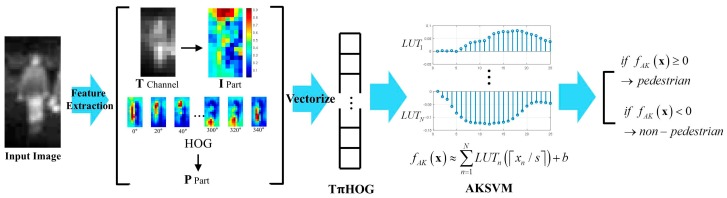
Test process of TπHOG-AKSVM for nighttime PD.

In this subsection, how to combine AKSVM with TπHOG for nighttime PD is explained. The test process of the combination of AKSVM with TπHOG is summarized in Figure 14.

As shown in the figure, the HOG and **T** channel are extracted from input IR image first. Then **P** and **I** parts are extracted from HOG and **T** channel, respectively. All these features are vectorized and TπHOG is completed by concatenating these vectorized features (**T** channel, **P** part, **I** part, HOG). Then, the score of each component in TπHOG is read off from the LUT and the total score of TπHOG is computed by summing the scores of the component features. Finally, if the total score is larger than 0, the input image is classified as pedestrian. Otherwise, it is classified as non-pedestrian.

## 4. Experimental Results

In this section, the proposed method is applied to the KAIST pedestrian dataset [14] and its performance is compared with other conventional methods. The KAIST pedestrian dataset consists of a number of pairs of visible-thermal images that are aligned in the image size of 640 × 512. The dataset images were taken by both visible and thermal sensors (FIR) in the day and nighttime at three locations (Campus, Road, Downtown). In this experiment, we use images in the nighttime of the KAIST dataset for training and testing. In the nighttime dataset, there are 838 training images with 1122 annotations and 797 test images.

In this experiment, we set the size of the ROI image to 64 × 32 pixels, the cell size of the HOG to 4 × 4 pixels, and the block size of the TπHOG to 4 × 4 cells. The AKSVM classifiers are trained with TπHOGs of training set using the LibSVM MATLAB toolbox [24,34]. For LUTs of AKSVM, we used the LUT of $N_{L}=100$ and s=0.01. Piotr’s Computer Vision Toolbox [35] is also used for feature extraction and testing.

**Figure 15:**
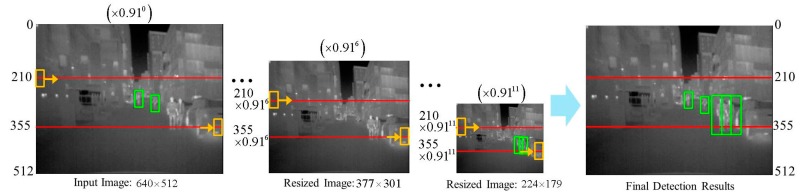
Example of detecting pedestrians of multiple scales using sliding window approach.

For testing, sliding window approach is employed to detect pedestrians with various scales. In the sliding window approach, the step size is fixed to the cell size and scale ratio is set to 1.09 (1/0.91), which result in 8 scales per octave. As in [36,37], we narrow down the search area by restricting the *y*-coordinate of the center of search window to lie within 210th and 355th pixel in *y*-axis. Shown in Figure 15 is an example of our sliding window approach for pedestrian detection. In the figure, the yellow boxes denote the search window, green boxes are the detection results and the red lines denote the boundaries of search region within which the *y*-coordinate of the center of search window is restricted.

We compare the detection performance of the proposed method with other conventional methods [10,19] using ROC curve and the log-average miss rate. The ROC curve shows the detection performance by plotting miss rate against the false positive per image (FPPI). The lower is the ROC curve, the better the detection performance. The log-average miss rate (MR) is the average of miss rate for FPPI of $\left[10^{-2},10^{0}\right]$ on the log scale. For comparison, we choose HOG-LinearSVM and the ACF-T-THOG [33] as a base line and the state-of-the-art, respectively. ACF-T-THOG utilizes pairs of visible-thermal images of nighttime and extracted ACF from visible images, T-THOG form thermal images. Except ACF-T-THOG, all SVM based classifier (LinearSVM, AKSVM) are trained with thermal images.

**Figure 16:**
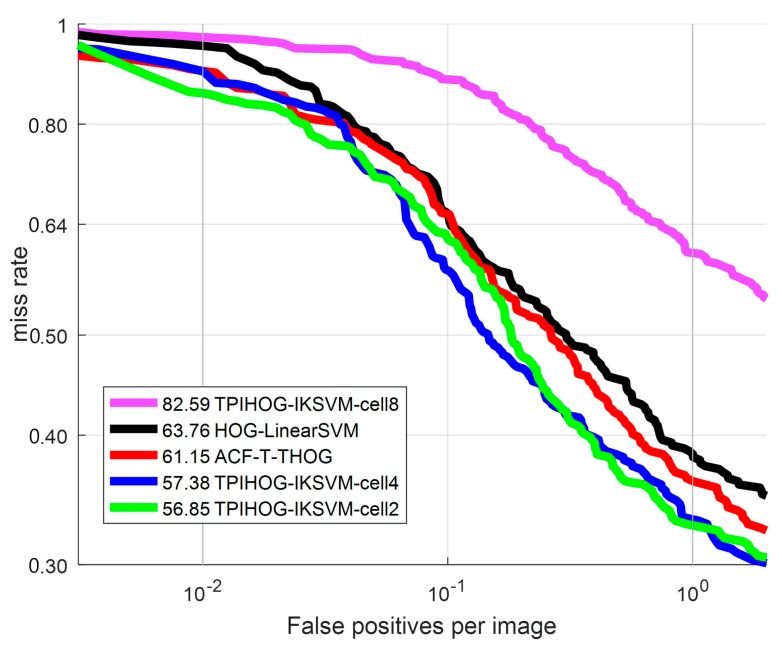
ROC curve: Comparison of classifiers with different cell size.

Then, the effect of the cell size on detection performance in analyzed. The ROC curves of TπHOG with AKSVM are plotted while varying the cell size in Figure 16. The intersection kernel $\kappa_{IK}$ in Equation (16) is used. The subsequent results (Log average MR, time per frame) are summarized in Table 1. In this experiment, we set the number of blocks for **P** part as 8 blocks per orientation layer as Figure 5.

As shown in Table 1 and Figure 16, the smaller the cell size of TπHOG is, the better detection performance is obtained. TπHOG-IKSVM-cell8 spends comparable detection time with ACF-T-THOG but it demonstrates about 30% higher Log average MR than ACF-T-HOG and HOG-LinearSVM. On the other hand, TπHOG-IKSVM-cell2 demonstrates the best performance among all the competing methods but its detection speed is about 20 times slower than TπHOG-IKSVM-cell4. Considering the trade-off between the detection performance (log average MR) and detection time, the recommended cell size is 4.

**Figure 17:**
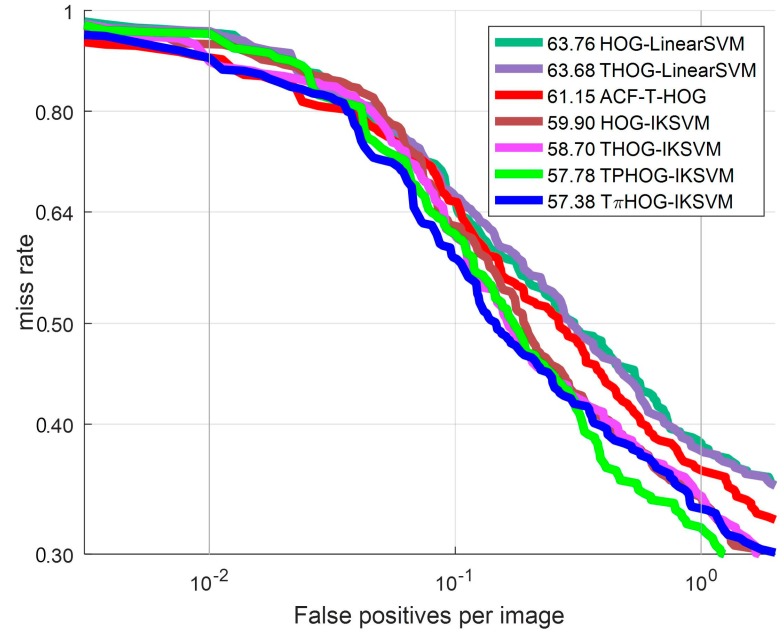
ROC curve: Comparison of feature.

To compare the discriminating power of the feature, we plot the ROC curves for several feature combinations with IKSVM in Figure 17. Table 2 presents the detailed information of the feature-classifier combinations used in experiments.

As shown in Figure 17, the THOG-LinearSVM performs better than the HOG-LinearSVM by 0.08% in terms of the log average MR. and THOG-IKSVM shows better detection performance than HOG-IKSVM by 1.2%. From this result, it can be observed that adding the **T** channel to the HOG improves detection performance from the conventional HOG. To examine the effect of the additive kernel, we measure the detection performance of the THOG-IKSVM. As shown in Figure 17 and Table 2, THOG-IKSVM demonstrates improved performance from THOG-LinearSVM indicating that the additive kernel resulted in significant improved detection performance of the SVM by 4.98% from the linear kernel.

Finally, we measure the detection performance using the TPHOG and the TπHOG in connection with the AKSVM. The TPHOG which adds the **P** part to THOG demonstrates 0.92% enhancement from THOG-IKSVM. The TπHOG which adds **I** part to TPHOG demonstrates improved performance from TPHOG by 0.4% and outperforms all other methods. From these experimental results, it can be seen that the TπHOG-AKSVM enhance the performance of conventional method in terms of both feature and classifier performance.

In terms of detection speed, the TπHOG-IKSVM takes more computational time than HOG-IKSVM by 1.06 s. To be specific, adding **P** part takes 0.8 s more time than HOG while **I** part and **T** channels requires additional 0.1 s compared with HOG.

**Figure 18:**
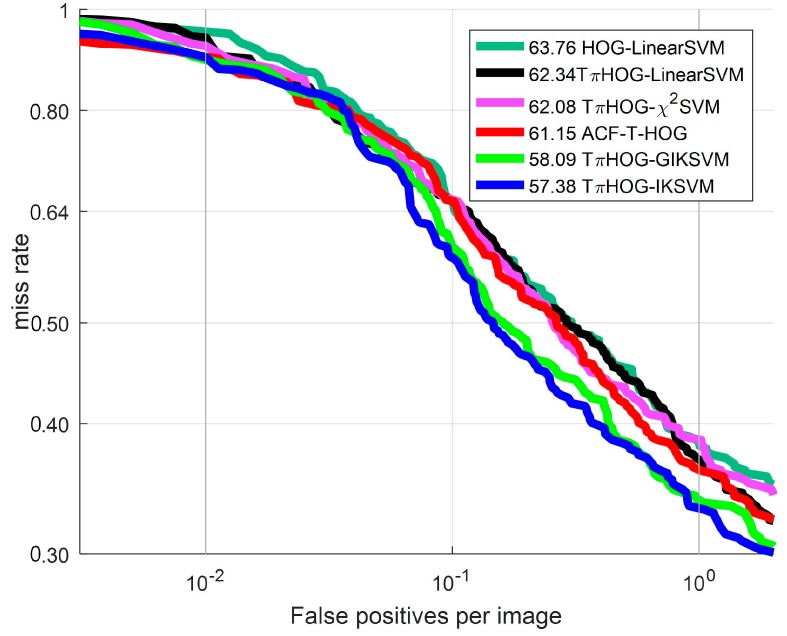
ROC curve: Comparison of additive kernel

Shown in Figure 18, shown are the ROC curves for the TπHOG with four different additive kernels: linear kernel, intersection kernel $\kappa_{IK}$, generalized intersection kernel $\kappa_{GIK}$ and $\chi^{2}$ kernel $\kappa_{\chi^{2}}$. The computational time of TπHOG-AKSVMs in Figure 16 are the same as one of TπHOG-IKSVM of Table 2 because they are performed with LookupTable of same size in this experiment.

**Figure 19:**
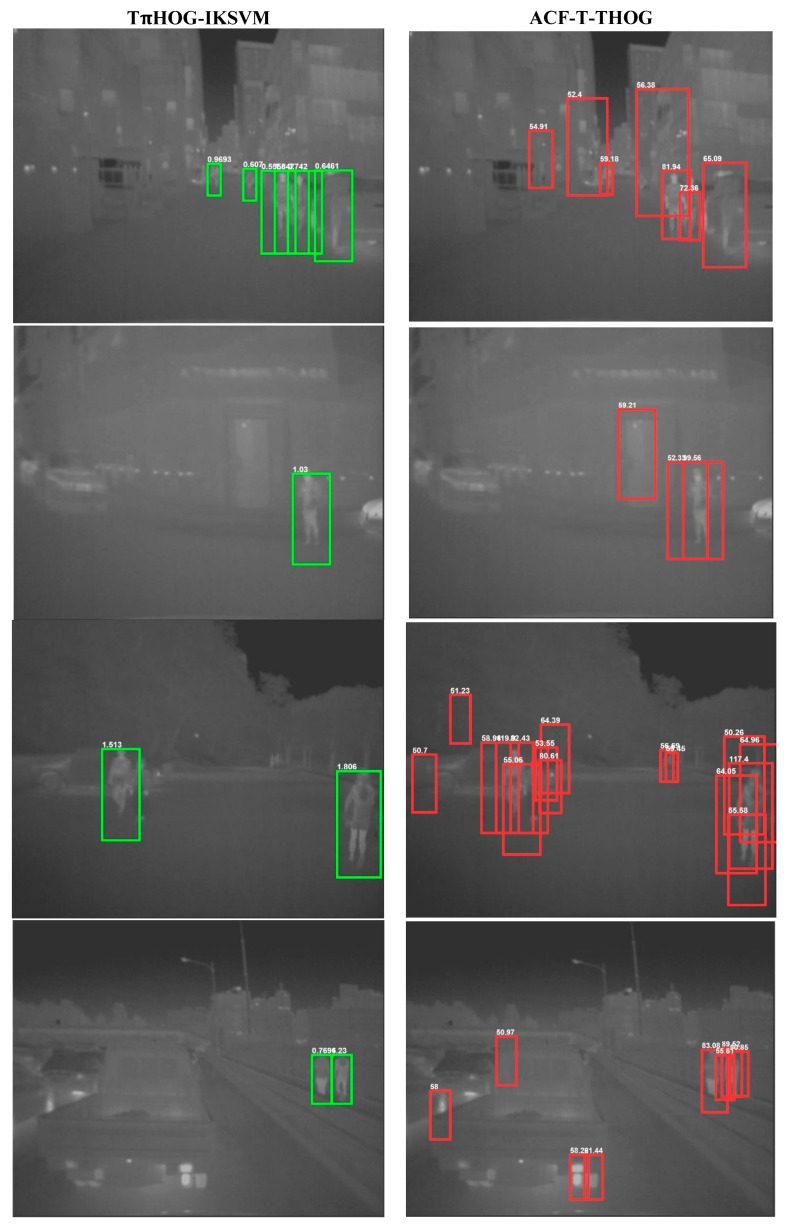
Detection results on test images of KAIST DB.

As shown in Figure 18, all types of TPIHOG-AKSVMs performs bettrer than the HOG-LinearSVM and TπHOG-LinearSVM. Among the AKSVMs, the TπHOG-IKSVM and TπHOG-GIKSVM demonstrate the significant improvement from the TπHOG-LinearSVM, and they also perform better than the ACF-T-HOG. In Figure 19, the examples of the detection results for TπHOG-IKSVM and ACF-T-THOG are compared

As shown in Figure 19, the ACF-T-THOG generates a lot of false positives to the vertical objects such as headlight or buildings. On the other hand, the proposed method shows good detection results with no false positive.

## 5. Conclusions

In this paper, a novel night-time pedestrian detection method using a thermal camera has been proposed. A new feature named TπHOG was developed and it was combined with AKSVM. The proposed TπHOG has more robust discriminative power than HOG because it uses not only the gradient information but also cell location of the gradient for each orientation channel. The proposed method was applied to KAIST pedestrian dataset and results show that its detection performance improved compared with other conventional methods for pedestrian detection in the nighttime. A comparison of experimental results with KAIST pedestrian dataset shows that the TπHOG performs better than the HOG in the distinctiveness of feature and the TπHOG-AKSVM shows better performance than other conventional methods.

## Figures and Tables

**Table 1:** Detailed information of feature-classifier.

	Feature	Cell Size	Log Average MR (%)	Time Per Frame (s)
ACF-T-HOG	ACF + T Channel + HOG	4	61.15	0.25
HOG-LinearSVM	HOG	4	63.76	1.78
TπHOG-IKSVM-cell8	TπHOG	8	82.59	0.29
TπHOG-IKSVM-cell4	TπHOG	4	57.38	2.84
TπHOG-IKSVM-cell2	TπHOG	2	56.85	39.28

**Table 2:** Detailed information of feature-classifier.

	Feature	Classifier	Log Average MR (%)	Time Per Frame (s)
ACF-T-HOG	ACF + **T** Channel + HOG	Decision Tree	61.15	0.25
HOG-LinearSVM	HOG	Linear SVM	63.76	1.78
HOG-IKSVM	HOG	AK SVM (Intersection Kernel)	59.90	1.78
THOG-LinearSVM	**T** Channel + HOG	Linear SVM	63.68	1.88
THOG-IKSVM	**T** Channel + HOG	AK SVM (Intersection Kernel)	58.70	1.88
TPHOG-IKSVM	THOG + **P** part	AK SVM (Intersection Kernel)	57.78	2.69
TπHOG-IKSVM	TPHOG + **I** part	AK SVM (Intersection Kernel)	57.38	2.84
